# Human–environmental overlap of resistant Enterobacterales: genomic evidence linking coastal waters and community carriage of antimicrobial resistance in a low- and middle-income setting

**DOI:** 10.3389/frabi.2025.1715797

**Published:** 2025-12-18

**Authors:** Appiah-Korang Labi, Noah Obeng-Nkrumah, Abigail Sarpong, Lady Asantewah Boamah Adomako, Christian Owusu-Nyantakyi, Rachel Ama Adadziwa Akorful, Mary-Magdalene Osei, Beverly Egyir, Japheth Awuletey Opintan

**Affiliations:** 1 Department of Medical Microbiology, University of Ghana Medical School, University of Ghana, Accra, Ghana; 2 Department of Medical Laboratory Sciences, School of Biomedical and Allied Health Sciences, University of Ghana, Accra, Ghana; 3 Environmental Biology Biotechnology and Health Division, Council for Scientific and Industrial Research, Water Research Institute, Accra, Ghana; 4 Bacteriology Department, Noguchi Memorial Institute for Medical Research, University of Ghana, Accra, Ghana

**Keywords:** one health, carbapenem-resistant Enterobacterales, wastewater, intestinal carriage, Ghana, antimicrobioal resistance (AMR)

## Abstract

**Background:**

Coastal waters contaminated by antimicrobial resistant hotspots may serve as reservoirs for third-generation cephalosporin resistant Enterobacterales (3GCR-E), extended-spectrum β-lactamase (ESBL)-producers, and carbapenem-resistant Enterobacterales (CRE), but their role in driving human carriage remains poorly understood.

**Aim:**

We investigated intestinal carriage of 3GCR-E, ESBL-producers, and CRE in coastal and inland communities in Accra, Ghana, and examined the genomic overlap between human and wastewater-derived CRE isolates.

**Methods:**

A comparative cross-sectional study was conducted from August 2023 to June 2024 with 800 participants (400 from coastal and 400 from inland communities). We cultured fecal samples from participants and water samples from lagoons and shorelines for 3GCR-E, ESBL-producers, and CRE. The CRE isolates from both human and wastewater were whole genome sequenced for comparison.

**Results:**

Overall, 53.6% (n=429/800) of participants carried 3GCR-E, with 43.6% being ESBL-producers and 1.5% being CRE, the latter restricted only to coastal residents. In the pooled analysis, inland residence was independently associated with reduced odds of 3GCR-E carriage (aOR 0.64, 95% CI 0.48–0.85; *p* = 0.001). For coastal participants, not swimming was protective against ESBL carriage (aOR 0.65, 95% CI 0.42–0.95; *p* = 0.030). All human and wastewater CRE isolates were *E. coli* and clustered in mixed-source phylogenetic clades (ST10, ST940) with >95% average nucleotide identity and pairwise SNP differences as low as 2–20. Both human and wastewater sources carried the identical carbapenemase gene *bla*OXA-181 on overlapping plasmid replicons, with 57–80% concordance across IncFIA, IncFIB (AP001918), IncX1, and Col440I.

**Conclusions:**

Our findings indicate a shared resistance gene pool between human and environmental sources, characterized by bidirectional CRE exchange but dominated by an environment-to-human transmission pathway. This underscores the urgent need for effective wastewater treatment and improved sanitation practices to reduce human exposure and curb the spread of antibiotic resistance.

## Introduction

The emergence and global spread of multidrug-resistant Enterobacterales — particularly third-generation-cephalosporin-resistant (3GCR-E), extended-spectrum β-lactamase-producing (ESBL-E), and carbapenem-resistant Enterobacterales (CRE) — pose a critical threat to public health (Noster et al., 2021). These pathogens are associated with limited treatment options, prolonged hospital stays, and high mortality rates (Antimicrobial Resistance Collaborators, 2022). Their proliferation is driven by both inappropriate antibiotic use in clinical settings and widespread environmental dissemination of resistance determinants.

In healthcare settings, 3GCR-E, ESBL-E, and CRE commonly spread through contaminated hospital environments, where they can persist on surfaces and colonize patients without causing immediate infection (Lykov and Volodkin, 2021; Noster et al., 2021). Such colonization often precedes invasive disease and facilitates silent transmission between patients, healthcare workers, and hospital wastewater systems. Although horizontal transmission within hospitals is well documented, the carriage burden also acts as an environmental amplification point, seeding resistant organisms into municipal drainage systems and water bodies through poorly treated wastewater (Manaia, 2017; Kumar and Pal, 2018). Consequently, community carriage — often initiated through environmental exposure — is increasingly recognized as a hidden reservoir that sustains the endemic presence of ESBL-E, 3GCR-E, and CRE within populations (Mughini-Gras et al., 2019).

Coastal waters represent particularly vulnerable ecosystems because they receive untreated sewage and wastewater from densely populated urban centers (Banu et al., 2021). Regular users of such environments, including fisherfolk and recreational swimmers, face heightened risks of colonization and subsequent infection with resistant bacteria (Leonard et al., 2018). The Korle-Lagoon in Accra, Ghana, is regarded as one of the most polluted coastal water bodies globally (Amankwaa et al., 2014). It receives wastewater from multiple sources, including Ghana’s largest tertiary hospital—the Korle-Bu Teaching Hospital—as well as major treatment facilities such as the Lavender Hill Fecal Sludge Treatment Plant and the Mudor Wastewater Treatment Plant (Banu et al., 2021; Tettey et al., 2024). Previous studies have documented high levels of antimicrobial-resistant Enterobacterales, including extended-spectrum β-lactamase producers, in tributaries feeding into the Korle-Lagoon (Banu et al., 2021; Tettey et al., 2024).

Despite this, the potential human health impact of environmental exposure to resistant organisms in such high-burden coastal ecosystems, especially in low-income settings, remains poorly understood (Larsson et al., 2023). Specifically, there are limited data comparing colonization risk among individuals living adjacent to coastal AMR hotspots (Donkor et al., 2024). In this study, we assessed the prevalence and risk factors for fecal carriage of 3GCR-E and ESBL-E in a coastal community affected by the Korle-Lagoon, compared with an inland community unexposed to this hotspot. We further compared human-derived CRE isolates with contemporary isolates from coastal waters to characterize carbapenemase genes, plasmid backbones, clonal lineages, and genomic overlap between these sources.

## Methods

### Study design and setting

**Figure 1:**
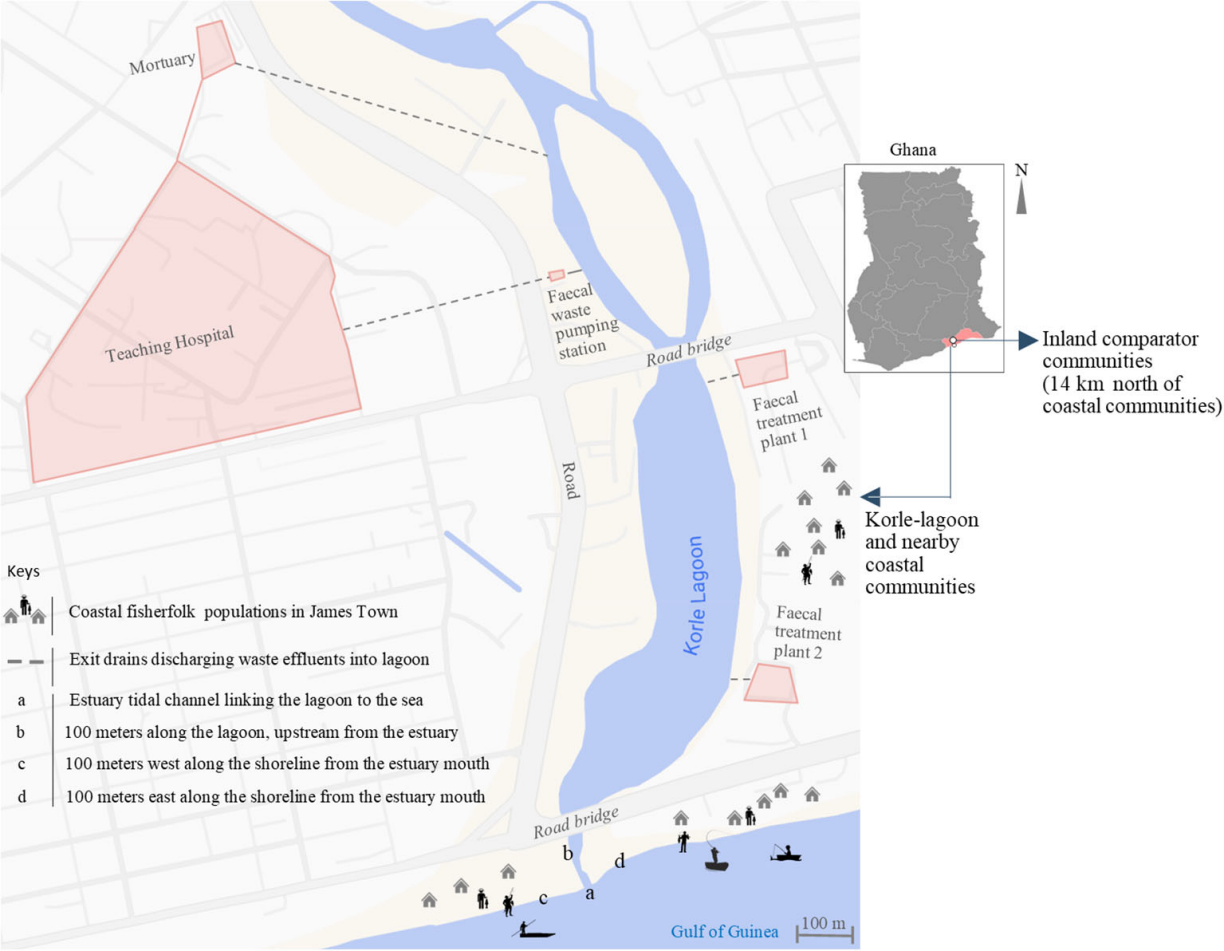
Map of the Korle-Lagoon and nearby coastal communities in James Town, Accra, showing sampling sites (a–d): (a) the estuary where the lagoon enters the sea, (b) 100 meters upstream of the estuary along the lagoon, (c) 100 meters to the west of the estuary along the coastline, and (d) 100 meters to the east of the estuary along the coastline. Major health and sanitation infrastructure are also indicated. The inset map of Ghana highlights the locations of the coastal and inland comparator communities in the Greater Accra Region.

From August 2023 to June 2024, we conducted a community-based comparative cross-sectional study in coastal and inland communities of the Greater Accra Region, Ghana. Field sample collection and initial microbiological culturing of both human stool and environmental water samples were conducted between August and November 2023. The subsequent months (December 2023 to June 2024) was devoted to downstream laboratory analyses, including molecular characterization and final data analysis. The coastal settlements are situated in James Town (Accra, Ghana) (Ayilu, 2023), a historic fishing hub with colonial-era structures dating back to the 17th century (**Figure 1**). The fisherfolks in James Town operate along the estuary where the Korle-Lagoon meets the sea and the adjoining coastline. These coastal sites are heavily exposed to untreated sewage and hospital effluents discharged through the lagoon into the marine environment. The inland comparison site was Ashongman (Accraba, 2025), a peri-urban community located approximately 14 km north of the coastal settlements. Although the site was purposively selected based on prior community engagement and logistical feasibility, it was also chosen because it shares similar demographic and socioeconomic characteristics with the coastal community. Both communities are located within the Greater Accra Region and are characterized by urban–peri-urban structures. Ashongman has an estimated population density of about 1,725 persons per square kilometer, while James Town has a higher density of approximately 12,000 persons per square kilometer. Despite this difference, both communities share similar sanitation practices and access to primary healthcare services, including nearby polyclinics and community pharmacies. The key distinction is the absence of direct exposure to the Korle-Lagoon ecosystem.

### Participant recruitment

The study population comprised all apparently healthy residents of the selected coastal and inland communities. We employed a two-stage sampling strategy. First, we used systematic random sampling to identify eligible households within each community. Next, within each selected household, we used the Kish method (Gaziano, 2008) to select a single, eligible respondent. To ensure cohort integrity and minimize follow-up loss, only participants aged ≥ 18 years were enrolled, as they were more likely to provide reliable data and stool samples. We excluded potential inland participants who had visited coastal waters within the past 3 years. After obtaining informed consent, a standardized questionnaire was administered to each participant. This instrument collected data on socio-demographics (age, gender, marital status, place of residence), lifestyle and hygiene practices (source of drinking water, handwashing habits, type of toilet facility, waste disposal methods), recent antibiotic use, hospitalization history, and other factors related to AMR risk. Participants were also asked about international travel, alcohol consumption, smoking status, and their practices for storing bathing water. Two types of environmental exposure were recorded in past one year: swimming in lagoon or nearshore marine waters, representing routine recreational or occupational water contact; and previous non-swimming interactions with lagoon or shoreline waters (e.g., fishing, trading, or washing equipment). All participants self-collected stool samples, which were transported on ice to the laboratory within two hours of collection.

### Water sampling

During the same period, wastewater samples were prospectively collected from multiple defined sites along the lagoon and adjoining coastline (**Figure 1**) for microbiological investigations. The sampling sites included 100 meters upstream of the estuary where the lagoon discharges into the sea, directly at the estuary, and 100 meters along the shoreline to the left and right of the estuary (**Figure 1**). Water samples were collected weekly for 4 months between August and November 2024. During each sampling event, 500 mL grab samples were collected from each of the four defined sites, placed into separate sterile bottles, and processed individually. All samples were immediately transported on wet ice to the laboratory and analyzed within 24 hours of collection.

### Microbiological investigations

Fecal samples were cultured on MacConkey agar (Becton Dickinson, USA) supplemented with 4 µg/mL of cefotaxime and on a separate MacConkey agar plate supplemented with 4 µg/mL of meropenem. All plates were incubated at 37°C for 24 hours. Putative 3GCR-E were isolated from the cefotaxime-supplemented plates, while presumptive carbapenem resistant bacteria were isolated from the meropenem-supplemented plates. The dominant Enterobacterales colonies from each selective agar were identified using Matrix Assisted Laser Desorption Ionization-Time of Flight Mass Spectrometry (MALDI-TOF MS) and subcultured for further analysis. Participants with a negative stool culture on MacConkey were excluded from further analyses. For wastewater, 100 mL aliquots of the grab sample from each site were serially diluted (1:10, 1:100, 1:1,000), and each dilution was concentrated separately by membrane filtration using 47 mm, 0.45 µm mixed cellulose ester membranes (Microdisc^®^, Membrane Solutions, USA). The membranes were then placed on Tryptone Bile X-Glucuronide (TBX) agar (Oxoid™, UK) supplemented with 4 µg/mL of meropenem and incubated at 37 ± 1 ^∘^C for 24 hours. Colonies exhibiting a characteristic blue–green coloration were counted as presumptive *Escherichia coli*, and results were expressed as colony-forming units (CFU) per 100 mL of wastewater. Counts were recalculated as CFU/mL for standardization. Representative *E. coli* colonies were confirmed by MALDI-TOF MS and preserved for downstream analysis.

#### Susceptibility tests for resistant mechanism

All presumptive 3GCR-E and meropenem resistant Enterobacterales from fecal and wastewater samples were subjected to antimicrobial susceptibility testing using the Kirby–Bauer disc diffusion method (CLSI, 2024) with ceftazidime (30 µg), cefotaxime (30 µg), and meropenem (30 µg). Results were interpreted according to Clinical Laboratory Standard Institute (CLSI) M100 Performance Standards for Antimicrobial Susceptibility Testing (M100). Isolates resistant to cefotaxime and/or ceftazidime were confirmed as 3GCR-E, while meropenem resistant isolates were classified as carbapenem resistant. ESBL production was confirmed by the combination disc method, defined as a ≥5 mm increase in inhibition zone diameter with clavulanic acid (M100). Meropenem resistant isolates were further examined using the modified carbapenem inactivation method (mCIM) (M100), with positive isolates confirmed as CRE. *Klebsiella pneumoniae* ATCC BAA-1705 was used as the carbapenemase-producing control, whereas ATCC BAA-1706 served as the non-carbapenemase-producing reference strain.

### Whole genome sequencing

Whole-genome sequencing of *Enterobacterales* isolates was performed using the Illumina MiSeq platform (Illumina, USA) with library preparation and sequencing conducted according to the manufacturer’s standard protocols. Genomic DNA was extracted from all CRE using the QIAamp DNA Mini Kit (Qiagen, Hilden, Germany), following the manufacturer’s protocol optimized for bacterial cells. The extracted DNA was quantified using a Qubit fluorometer (Thermo Fisher Scientific, USA). Sequencing libraries were prepared using the Illumina DNA Prep – (M) Tagmentation Kit (Illumina, USA), which utilizes bead-linked transposomes for simultaneous fragmentation and adapter ligation. Library quality and fragment size distribution were evaluated using a Bioanalyzer (Agilent Technologies, USA) to confirm appropriate insert sizes for sequencing. Indexed libraries were pooled equimolarly and loaded onto the Illumina Nextseq 1000 platform (Illumina Inc., USA), using a paired-end sequencing strategy with read lengths of 2 × 150 base pairs.

#### Bioinformatic analysis

Bioinformatics analyses were performed using an in-house pipeline according to broad standards outlined in the WHO GLASS protocol for WGS and the European Commission Health and Digital Executive Agency guidelines (GLASS whole-genome sequencing for surveillance of antimicrobial resistance; European Commission, 2022). Briefly, the quality of the raw reads was assessed using FASTQC and multiQC (Babraham Bioinformatics - FastQC A Quality Control tool for High Throughput Sequence Data; Ewels et al., 2016). Trimmomatic was used to remove adaptor sequences and reads with low quality (Bolger et al., 2014). Trimmed reads were then assembled using the spades optimizer Unicycler (Wick et al., 2017). Resistance genes were identified with Abricate (https://github.com/tseemann/abricate) using the ResFinder and CARD databases (Alcock et al., 2020; Bortolaia et al., 2020), virulence genes with VirulenceFinder (Joensen et al., 2014), and mobile elements with PlasmidFinder (Carattoli et al., 2014). Comparative genomics was performed on the Centre for Genomic Epidemiology platform (https://www.genomicepidemiology.org/services/) to determine multilocus sequence types (MLST) and single nucleotide polymorphisms (SNPs). Phylogenies were generated with CSIPhylogeny, aligned to reference strain NZ_LR134222.1, and visualized in iTOL (Letunic and Bork, 2021) with resistance and virulence gene annotations. Average Nucleotide Identity (ANI) was calculated with fastANI to confirm species-level assignments. Genomic diversity across isolates was assessed using Simpson’s Index of Diversity (1–D). Overlap was quantified using the Jaccard similarity index, calculated as the number of shared resistance genes or plasmid replicons divided by the total number of unique features across human and wastewater isolates.

### Data availability

The assembled genomes of the isolates in this study are available in the NCBI GenBank under bioproject accession number PRJNA1297679.

### Statistical analysis

Data was analyzed using STATA version 16 (StataCorp, College Station, TX, USA). Carriage rates were estimated for each community as the proportion of colonized individuals relative to sample size. Categorical variables were compared using the χ² test, while continuous variables were assessed using Student’s *t*-test or the Mann–Whitney *U* test, as appropriate. Independent predictors of risk for carriage of 3GCR-E and ESBL-producing Enterobacterales were evaluated using multivariable logistic regression and results are presented as adjusted odds ratios (aORs) with 95% confidence intervals (CIs). Model calibration was assessed with the Hosmer–Lemeshow goodness-of-fit test (*p* > 0.05 indicating adequate fit). Statistical significance was defined as a two-tailed *p* < 0.05. Box-and-whisker plots were used to display the median, interquartile range (IQR), and whiskers representing the minimum and maximum values within 1.5× IQR.

### Ethical considerations

The study received ethical approval from the Ghana Health Service Ethics Review Committee with IRB number-GHS-ERC-006/08/22. Signed informed consent was obtained from all participants.

## Results

A total of 821 participants were initially recruited for this study, with 410 individuals drawn from coastal communities and 411 from inland communities. Of these, 21 participants provided interview data but were unable to submit stool specimens; as a result, their data were excluded from the analysis. The final dataset comprised 800 participants (n=400 per community) who provided both stool specimens and completed questionnaires, resulting in a 97.4% completion rate of the recruited cohort. Sociodemographic characteristics of the study participants are summarized in **Supplementary Data 1**. None of the inland participants reported prior contact with the Korle-Lagoon or its estuary, whereas all coastal participants reported previous exposure to the lagoon waters or its estuary. Swimming was significantly prevalent among coastal residents, with 90% (n=360/400) having swam in the past year, compared to only 10% (n=40/400) of inland participants. In terms of health behaviors, approximately 55% (n=221/400) of inland respondents reported antibiotic use in the past year, compared to 27% (n=108/400) of coastal participants.

### Prevalence of intestinal carriage of 3GCR-E

Among the 800 participants, 429 (53.9%) were colonized with 3GCR-E, comprising 236 (59.0%) from the coastal cohort and 193 (48.3%) from the inland group (χ² = 8.87, *p* = 0.003) (**Table 1**). Extended-spectrum β-lactamase-producers (n = 349; 43.6%) were identified in 188 (47.0%) coastal and 161 (40.3%) inland participants (χ² = 3.44, *p* = 0.064). Carbapenem-resistant Enterobacterales were detected exclusively among coastal participants (n = 12/400; 3.0%) and were absent among inland participants (n = 0/400) (χ² = 10.24, *p* = 0.001). All 12 CRE isolates co-expressed ESBLs, and *E. coli* was the sole species identified among all CRE cases. Colonization with other 3GCR-E lacking both ESBL and carbapenem resistance was more frequent among coastal participants (n = 48/400; 12.5%) than among those from inland communities (n = 32/400; 8.0%) (χ² = 3.13, *p* = 0.077). *Escherichia coli* was the predominant species overall, accounting for 99% of 3GCR-E and ESBL isolates in the coastal cohort and approximately 96% in the inland cohort.

**Table 1:** Intestinal colonization with 3rd-generation cephalosporin-resistant, ESBL-producing, and carbapenem-resistant Enterobacterales in coastal and inland cohorts.

Total participants	All participants (n=800, %)				Coastal cohort (n=400, %)				Inland cohort (n=400, %)			
	3GCR-E	ESBL	CRE	*Oth	3GCR-E	ESBL	CRE	*Oth	3GCR-E	ESBL	CRE	*Oth
All Enterobacterales	429(53.6)	349(43.6)	12(1.5)	80(10.0)	236(59.0)	188(47.0)	^#^12 (3.0)	48(12.5)	193(48.3)	161(40.3)	0	32(8.0)
*E. coli*	419(97.7)	343(98.2)	12(100)	76(95.0)	234(99.1)	187(99.4)	^#^12(100)	47(98.0)	185(95.9)	156(96.9)	0	29(90.6)
*K. pneumonaie*	7(1.6)	4(1.2)	0	3(3.7)	0	0	0	0	7 (3.6)	4(2.4)	0	3(9.4)
*E. hormachei*	1(0.2)	1(0.3)	0	0	0	0	0	0	1 (0.5)	1(0.6)	0	0
*C. freundii*	1(0.2)	0	0	1(1.3)	1(0.4)	0	0	1(2.1)	0	0	0	0
*Salmonella* sp.	1(0.2)	1(0.3)	0	0	1(0.4)	1(0.5)	0	0	0	0	0	0

3GCR-E, third-generation cephalosporin-resistant Enterobacterales; ESBL, extended-spectrum-beta-lactamases; CRE, carbapenem-resistant Enterobacterales. *Oth, other Enterobacterales that were resistant to third-generation cephalosporins but not ESBL-producers or carbapenem resistant.

^#^All 12 CRE were also ESBL-producers. Species percentages are calculated using counts for all Enterobacterales

### CRE carriage and exposure patterns

Among the 12 participants carrying CRE, the median age was 34 years (IQR 28–46), and 9 were females. The small number of CRE-positive participants precluded reliable multivariable modelling; however, descriptive analyses indicated consistent exposure patterns. In univariable analysis (**Supplementary Data 2**), all CRE carriers reported swimming or wading in lagoon or near-shore marine waters, compared with 89.5% (n=348/388) of CRE-negative residents (Fisher’s exact *p* = 0.619). Similarly, all CRE carriers had remained within their local community over the preceding year, whereas 17.8% (n=69/388) of CRE-negative residents reported travel outside the locality (*p* = 0.136), and 2.8% (n=11/388) had travelled outside Ghana (*p* = 1.000). None of the CRE-positive participants reported hospital admission within the past year, compared with 7.5% (n=30/388) of CRE-negative residents (*p* = 0.612). Although none of these associations reached statistical significance, the consistent patterns suggest that CRE colonization in this cohort may be predominantly community-acquired, associated with frequent contact with lagoon and shoreline waters, rather than imported or healthcare-related exposure.

### Risk factors for intestinal carriage of 3GCR-E and ESBL-producers

**Table 2** summarizes the multivariable logistic regression models identifying risk factors for intestinal carriage of 3GCR-E and ESBL-producers, stratified by study cohorts. Among all participants (pooled analysis, n=800), 3GCR-E carriage was independently associated with female sex (aOR 1.45, 95% CI 1.08–1.95) and use of tap water as the primary drinking water source (aOR 1.44, 95% CI 1.13–1.96). Individuals with no swimming exposure (aOR 0.61, 95% CI 0.45–0.84) and no hospital admission in the past year (aOR 0.15, 95% CI 0.06–0.41) were significantly less likely to carry 3GCR-E, while residence in inland communities was protective (aOR 0.64, 95% CI 0.48–0.85). In coastal cohorts, waste disposal by pit dumping and burning was also protective (aOR 0.30, 95% CI 0.12–0.75). For ESBL-E, neither gender nor cohort type was a significant predictor of intestinal carriage. Risk was instead associated with use of tap water (pooled analysis: aOR 1.55, 95% CI 1.15–2.05; coastal cohort: aOR 1.50, 95% CI 1.05–2.20), while absence of swimming remained protective across cohorts (pooled analysis: aOR 0.70, 95% CI 0.50–0.95). Hospital admission was the most consistent predictor of ESBL-E carriage, with substantially lower odds among participants with no prior admission in both coastal (aOR 0.18, 95% CI 0.07–0.48) and inland (aOR 0.25, 95% CI 0.10–0.65) cohorts. 3,4,4,5,5,5,6,

**Table 2:** Multivariable logistic regression analysis of factors associated with intestinal carriage of third-generation cephalosporin-resistant and ESBL-producing Enterobacterales.

Factors	Carriage with 3GCR-E						Carriage with ESBL-producing Enterobacterales					
	All participants		Coastal cohort		Inland cohort		All participants		Coastal cohort		Inland cohort	
	aOR (95%CI)	*P*	aOR (95%CI)	*P*	aOR (95%CI)	*P*	aOR (95%CI)	*P*	aOR (95%CI)	*P*	aOR (95%CI)	*P*
Gender: Male	1.45 (1.08–1.95)	0.014	–		–		–		–		–	
Mode of waste disposal: Dumped in pits and burnt	-		0.30 (0.12–0.75)	0.010	-		-		0.50 (0.25–0.95)	0.030	-	
Source of drinking water: Tap	1.44 (1.13–1.96)	0.017	–		–		1.55 (1.15–2.05)	0.003	1.50 (1.05–2.20)	0.020	-	
Swimming in any waterbody the past year: No	0.61 (0.45–0.84	0.002	-		0.82 (0.43–0.91)	0.002	0.70 (0.50–0.95)	0.020	0.65 (0.42–0.95)	0.030	0.80 (0.64–0.99)	0.048
Hospital admission: No	0.15 (0.06–0.41)	0.002	–		–		0.20 (0.08–0.50)	0.001	0.18 (0.07–0.48),	0.001	0.25 (0.10–0.65)	0.004
Cohort type: Inland community	0.64 (0.48–0.85)	0.001	-		-		-		-			

aOR, adjusted Odds Ratio; CI, confidence interval; *p*, p-value; 3GCR-E, third-generation cephalosporin resistant Enterobacterales; ESBL, extended-spectrum beta-lactamase.

### Carbapenemase genes in CRE from coastal cohorts

**Figure 2:**
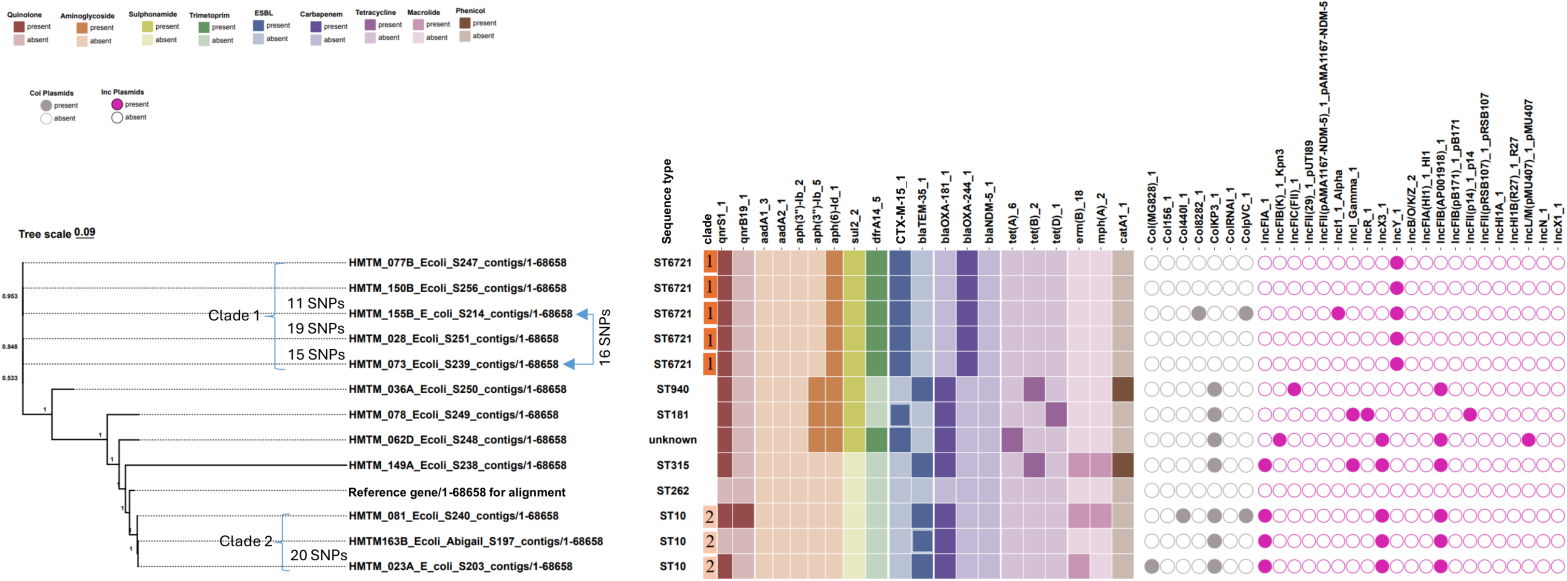
Whole-genome phylogenetic clustering of carbapenemase-producing *E. coli* isolates from human coastal cohorts showing associated resistance genes and plasmid replicons. The figure displays 13 entries representing the 12 study isolates together with one reference genome (reference/1-68658) used for sequence alignment and SNP calling. SNP, single nucleotide polymorphism differences between strains (only SNP differences ≤ 30 are shown).

All 12 CRE isolates from humans harbored a carbapenemase gene, exclusively of the *blaOXA* family (**Figure 2**). Seven isolates belonging to diverse sequence types — ST10 (n = 3), ST181 (n = 1), ST315 (n = 1), ST940 (n = 1), and one unassigned ST — harbored the carbapenemase gene *blaOXA-181*. Five of these also carried the ESBL gene *blaTEM-35*, whereas the remaining two co-harbored *blaCTX-M-15* ESBL gene. The other five CRE isolates, all assigned to ST6721, carried the carbapenemase gene *blaOXA-244* together with *blaCTX-M-15* ESBL. Plasmid replicon profiling revealed non-random associations between carbapenemase type and plasmid backbone, suggesting distinct vehicles for the dissemination of *blaOXA-181* and *blaOXA-244*. Specifically, all ST6721 CRE carrying *blaOXA-244* together with *blaCTX-M-15* were associated with a conserved incompatibility plasmid backbone, IncY_1. Only one of these isolates additionally demonstrated co-residence of multiple plasmid types within the same host, harboring colicinogenic plasmids (Col8282 and ColpVC) alongside an IncI1_Alpha replicon. In contrast, *blaOXA-181* was distributed across five distinct sequence types and mobilized across a more heterogeneous plasmid repertoire (median, 5 replicons per isolate; range, 3–6; Mann–Whitney U test, *p* = 0.032). The seven *blaOXA-181*–carrying isolates, which also harbored either *blaCTX-M-15* or *blaTEM-35*, contained diverse combinations of Col- and Inc-type plasmid replicons. The ColKP3 plasmid type was found in all *blaOXA-181* isolates. The InCFIB(AP001918) was present in 6 of 7 *blaOXA-181* isolates and in none of the *blaOXA*-244 isolates (Fisher’s exact test, *p* = 0.015). IncX3 was present in 5 of 7 *blaOXA-181* isolates and in none of the *blaOXA*-244 isolates (*p* = 0.028). Similarly, IncFIA_1 was detected in 4 of 7 *blaOXA-181* isolates but was absent among all *blaOXA-244* carriers (*p* = 0.081).

### Phylogeny of CRE from coastal cohorts

Phylogeny resolved the 12 CRE into two major clades (C1 and C2) and four singletons (**Figure 2**). Clade 1 comprised five isolates, all ST6721, with strong bootstrap support (100%). Four isolates (HMTM_028, HMTM_073, HMTM_150B, HMTM_155B) differed by ≤20 SNPs, consistent with recent clonal expansion, whereas HMTM_077B was more distantly related (>20–000 SNPs) within the C1-ST6721 lineage. Clade 1 exhibited low plasmid replicon diversity (Simpson’s index = 0.13), with all five ST6721 isolates sharing an identical resistome and a conserved IncY_1 plasmid backbone. Secondary colicinogenic plasmid acquisition within this lineage was limited; only one isolate carried additional replicons, comprising (Col8282 and ColpVC) and an IncI1_Alpha plasmid. Clade 2 comprised three isolates (HMTM_081, HMTM_163B, and HMTM_023A), all assigned to ST10, and displayed a resistome distinct from Clade 1. The isolates shared a conserved plasmid incompatibility backbone characterized by IncFIA_1, IncX3, and InCFIB(AP001918) replicons, while differing in their combinations of colicinogenic plasmids. Diversity analysis confirmed moderate plasmid variation (Simpson’s index = 0.32). The SNP analysis suggested a recent clonal relationship between HMTM_163B and HMTM_023A (20 SNPs), while HMTM_081 was more distant (>250 SNPs). Outside Clades 1 and 2, four singleton isolates (ST940, ST315, ST181, and one with an unassigned ST) exhibited heterogeneous resistomes and non-overlapping plasmid replicon combinations (Simpson’s index: ST diversity = 0.75; plasmid diversity = 0.63).

### Occurrence of CRE in coastal water bodies

**Figure 3:**
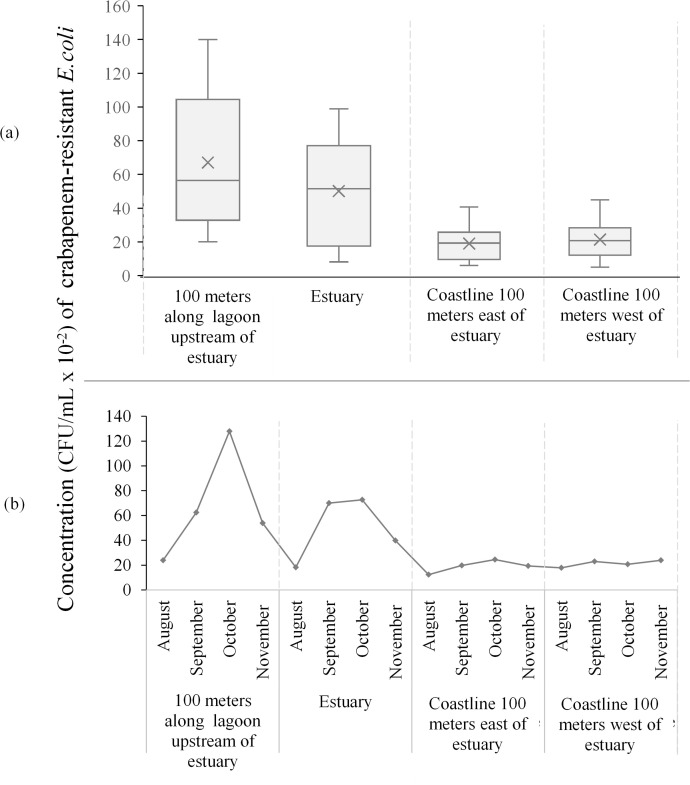
Relative abundance of carbapenem-resistant *E. coli* expressed in colony forming units per milliliters (CFU/mL) at wastewater sampling points along the Korle-Lagoon and adjoining coastline. For visualization, y-axis values are scaled down by 10² (axis label ‘CFU/mL × 10⁻²’). Actual counts are obtained by multiplying plotted values by 10². (a) Box-and-whisker plots illustrating spatial variation in CRE distribution, showing means, medians, interquartile ranges, and full concentration ranges across sampling points. (b) Trends in average CRE concentrations from August to November 2024 across sampling locations.

Because residence in coastal communities was identified as a significant risk factor for intestinal colonization with 3GCR-E — and CRE (all *E. coli*) carriage was observed exclusively among coastal participants — we collected water samples from coastal water bodies to investigate potential environmental sources of CRE exposure. A total of 64 water samples were cultured for carbapenem-resistant *E. coli*, comprising 16 samples collected at each of four sampling points (**Table 3**). **Figure 3a** illustrates the concentration of CRE along the wastewater pathways. Carbapenem-resistant *E. coli* were detected in 96.8% (n = 62/64) of composite grab samples. The CRE concentrations were highest in the lagoon upstream of the estuary (median = 5,700 CFU/mL; range up to 14,000 CFU/mL), decreasing at the estuary itself (median = 4,200 CFU/mL; maximum 10,000 CFU/mL). In contrast, CRE concentrations were consistently detected along the adjoining open shorelines, indicating a stable environmental reservoir throughout the sampling period. These concentrations, however, were substantially lower (median = 2,000 CFU/mL; range = 1,000–4,000 CFU/mL) than those found at the estuary and along the lagoon (**Figure 3b**).

**Table 3:** Prevalence and concentrations of carbapenem-resistant *E. coli* across coastal water sampling sites.

Measure	Sampling sites				All sites
	Along lagoon 100 m before estuary	Estuary	Coast sea water 100 m to the right of estuary	Coast sea water 100 m to the left of estuary	
Prevalence	100% (n=16/16)	100% (n=16/16)	93.75% (n=15/16)	93.75%(n=15/16)	96.75 (n=62/64)
Median in CFU/100 mL	5640	5152.5	1926	2075	2440
Range (Min-Max) in CFU/100 mL	2000-14001	811-9890	700-4967	510-4500	510-14001

The equivalent sample volume analyzed was 100 mL corresponding to a detection limit of 1 CFU/100 mL; Min, minimum; Max, maximum.

### Molecular characterization of CRE from wastewater samples

**Figure 4:**
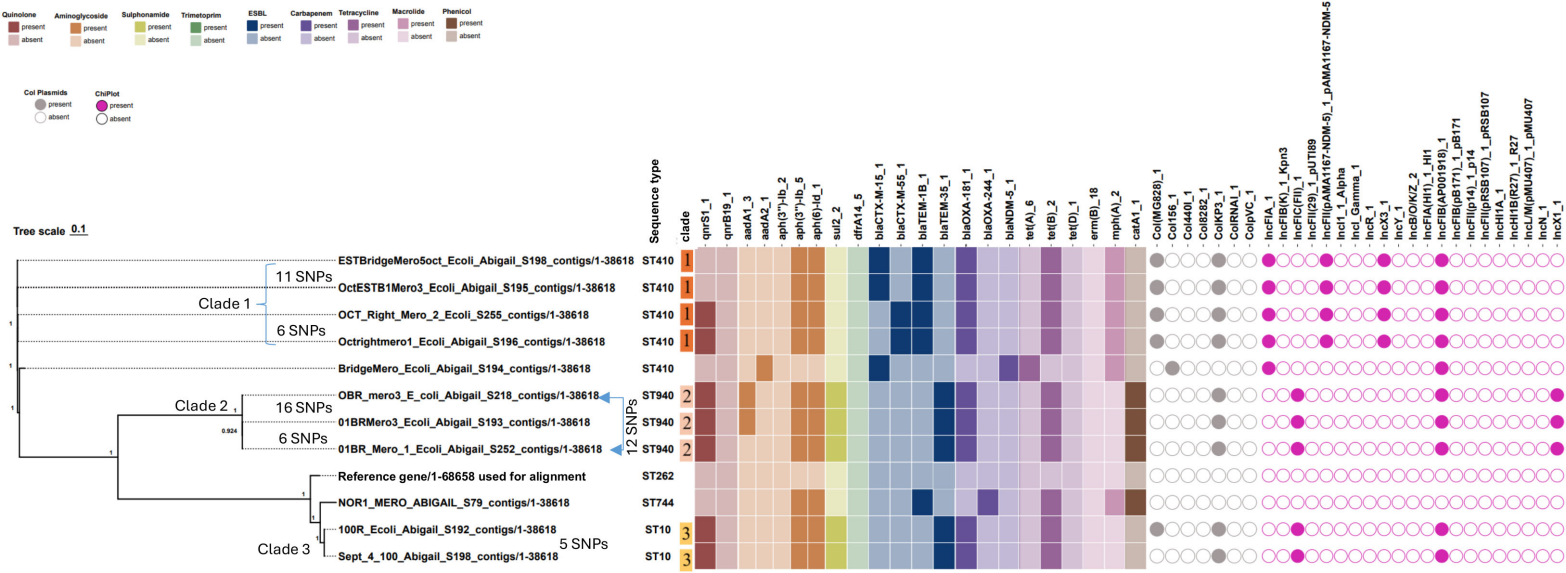
Whole-genome phylogenetic clustering of carbapenemase-producing *E. coli* isolates from water isolates showing associated resistance genes and plasmid replicons. The figure displays 12 entries representing the 11 study isolates together with one reference genome (reference/1-68658) used for sequence alignment and SNP calling. SNP, single nucleotide polymorphism differences between strains (only SNP differences ≤ 30 are shown).

We randomly selected 11 CRE recovered from wastewater samples for WGS. All CRE isolates carried a corresponding carbapenemase gene (*blaOXA-181*, n = 9; *blaOXA-244*, n = 1; *blaNDM-5*, n = 1) and harbored one or two combinations of ESBL genes (*blaCTX-M-15*, n = 3; *blaCTX-M-55*, n = 2; *blaTEM-35*, n = 5) (**Figure 4**). Maximum-likelihood phylogeny resolved the isolates into three distinct clades (C1–C3), with one additional ST410 isolate (BridgeMero_Ecoli) carrying *blaNDM*-5 branching independently (**Figure 4**). Clade 1 comprised four ST410 isolates, all harboring *blaOXA-181* together with either *blaCTX-M-15* (n=2) or *blaCTX-M-55* (n=2) and exhibiting highly similar resistome profiles (pairwise Jaccard similarity ≈ 0.93–0.95) and plasmid backbones (Simpson diversity = 0.00) consistent with a conserved clonal lineage. All four Clade 1 isolates uniformly carried the plasmid incompatibility groups IncX3, IncFII(pAMA), IncFIA, and IncFIB(AP001918), together with the colicin-associated plasmids Col156 and Colkp3. Pairwise SNP analysis revealed that OctESTB1Mero3_S195 and ESTBridgeMero5oct_S198 differed by only 11 SNPs, while OCT_Right_Mero_2_S255 and Octrightmero1_S196 were separated by 6 SNPs. Clade 2 consisted of three ST940 isolates, each co-harboring *blaTEM*-35 ESBL and *blaOXA*-181 carbapenemase gene. All members of this clad shared the same plasmid repertoire comprising the incompatibility group IncFIC, IncFIB(AP001918), and IncX1 and the colicinogenic plasmid Colkp3. Only 6 SNPs separated 01BRMero3_S193 and 01BR_Mero_1_S252, and OBR_mero3_S218 differing by 12–16 SNPs from the other two. Clade 3 represented a more heterogeneous branch, comprising a single ST744 isolate carrying *blaOXA*-244 and two ST10 isolates co-harboring *blaTEM*-35 and *blaOXA*-181. The closest genomic relationship within Clade 3 was observed between Sept_4_100_S198 and 100R_S192, which differed by only 5 SNPs.

### Carbapenemase types and associated plasmid diversity in human and water isolates

Three carbapenemase types were detected across human and water sources: *blaOXA-181* predominated (n = 16/23; 9 water, 7 human), followed by blaOXA-244 (n = 6/23; 5 human, 1 water), and *blaNDM-5* in one water isolate. Among *blaOXA-181*–positive isolates, four distinct Col-type plasmid replicons were identified—two among water isolates and four across human isolates. ColKP3 was predominant and conserved across all 16 *blaOXA-181* isolates (7 human, 9 water), followed by ColMG826, found in five water and one human isolate. Eleven incompatibility plasmid types were detected overall (up to eight in human and six in water isolates). Among the 16 ColKP3 carriers, 15 also harbored IncFIB(AP001918) alongside IncFIA (n = 9), IncX3 (n = 9), and IncFIC(FII) (n = 7), indicating a conserved ColKP3 and IncFIB backbone with variable accessory plasmid content.

### Similarity of wastewater and human CRE isolates in coastal cohorts

**Figure 5:**
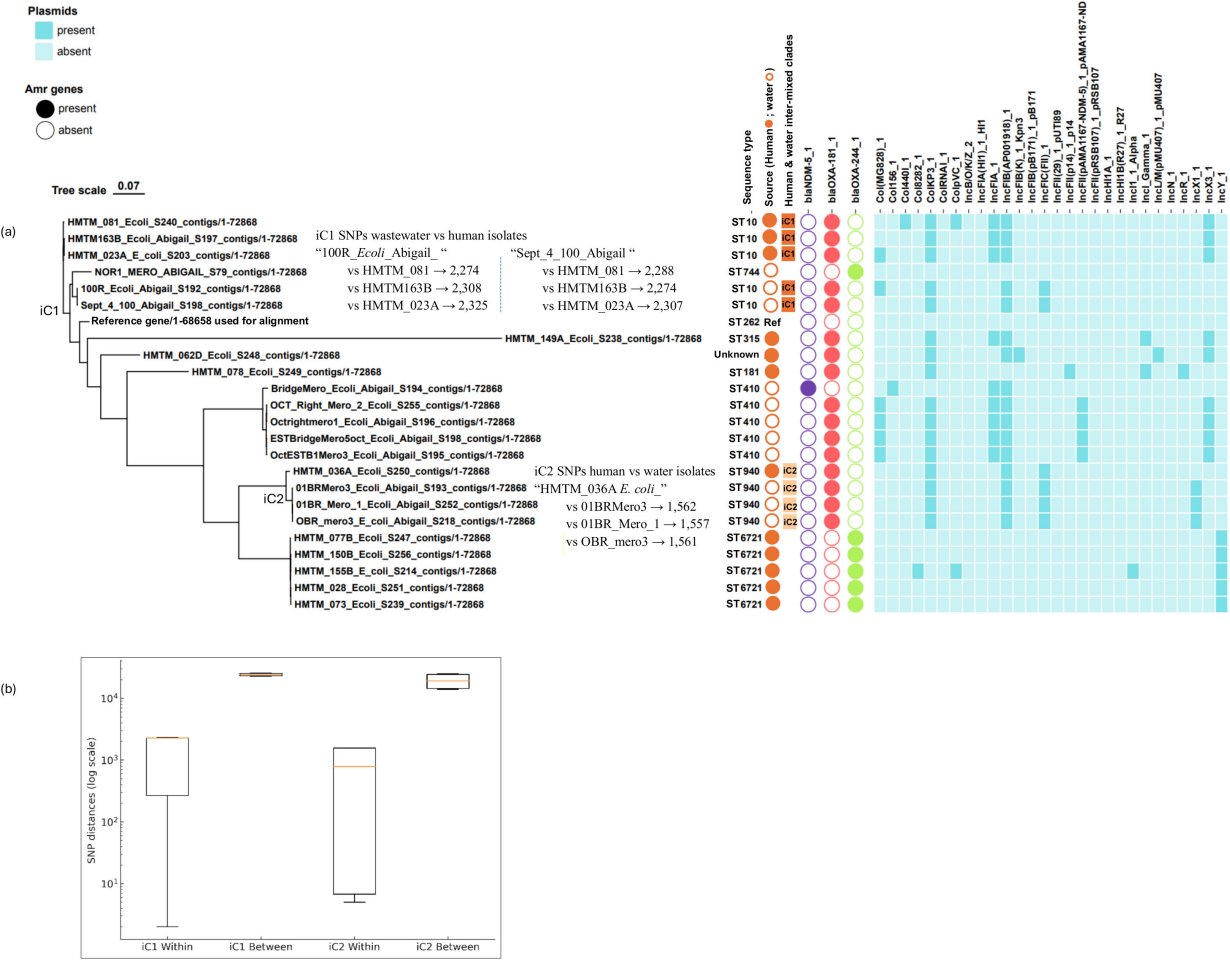
(a) Integrated phylogeny of human (HMTM) and water *E. coli* isolates showing clade structure, carbapenemase genes, and plasmid replicons. Human isolates are labelled HMTM; water isolates carry other labels. iC1, intermixed-source clade 1; iC2, intermixed-source clade 2; SNP, single nucleotide polymorphisms. The figure displays 24 entries representing the 23 study isolates together with one reference genome (reference/1-68658) used for sequence alignment and SNP calling. (b) Boxplot of SNP distances comparing within- and between-clade variation for iC1 and iC2. “Within” refers to pairwise SNP distances among isolates inside the same clade (e.g., all isolates in iC1 compared with each other, or all isolates in iC2 compared with each other). “Between” refers to pairwise SNP distances between isolates in iC1 or iC2 and those outside these clades. The log scale highlights the markedly lower within-clade SNP distances compared with between-clade distances.

Whole-genome maximum-parsimony phylogenetic analysis identified two inter-mixed human–wastewater lineages in which identical sequence types from different sources resolved into unified clades (iC1 and iC2) with > 95% bootstrap replicates (**Figure 5a**). In iC1, the ST10 lineage comprised three human isolates (HMTM_081, HMTM163B, HMTM_023A) and two wastewater isolates (100R_Ecoli, Sept_4_100). Pairwise SNP differences within this clade ranged from 2 to 2,325 SNPs (median 2,274; IQR 266–2,275), which were significantly lower than the >20,000 SNPs typically separating distinct ST lineages (median 24,010; IQR 22,953–25,186; Mann–Whitney U test *p* = 1.2 × 10⁻^7^) (**Figure 5b**). Average nucleotide identity (ANI) within iC1 was 99.2% (range 99.0–99.3%), compared with 94.8% (92.3–96.1%) between iC1 and other sequence types. In iC2, one human isolate (HMTM_036A) clustered with three wastewater isolates (01BRMero3, 01BR_Mero_1, OBR_mero3), all belonging to ST940. Within this clade, SNP differences ranged from 5 to 1,562 SNPs (median 783; IQR 7–1,559), again significantly lower than between-clade distances (median 19,015; IQR 14,317–24,276; Mann–Whitney U test *p* = 2.5 × 10⁻^5^). The ANI within iC2 was 99.3% (99.1–99.4%), compared to 94.9% (92.1–96.5%) between iC2 and other clades.

Within the iC1 (ST10) lineage, all three human and two water isolates carried the *blaOXA-181* gene on ColKP3-type colicinogenic plasmids, which co-existed with IncFIB(AP001918)-type incompatibility plasmids in the same isolates. In addition, the ColMG8288 plasmid type was detected in one human and one water isolate. Altogether, three of seven plasmid replicon types identified within iC1 were shared between human- and water-derived isolates, representing an inter-source plasmid overlap of 43% (n=3/7). Within iC2 ST940 lineage, all 3 water isolates and the human isolates carried *blaOXA-181* carbapenemase gene borne on ColKP3-type plasmids associated with IncFIB(AP001918) and IncFIC(F11) incompatibility plasmids (iC2 inter-source plasmid overlap 80%, n=3/4). Outside the mixed-source clades, human ST6721 isolates carrying *blaOXA-244* were dominated by IncY-type plasmid, which were absent among wastewater isolates (plasmid overlap: 0%, n = 0/11). In contrast, both human and water isolates harboring *blaOXA-181* but belonging to different sequence types all carried ColKP3-type plasmids, accompanied by up to 10 different plasmid types that varied between the two sources. Overall, within-clade inter-source plasmid overlaps were substantial (57–80%), whereas cross-clade overlaps were minimal (0-14%).

## Discussion

In this study, fecal carriage of 3GCR-E, including ESBL-producers, was high in both inland and coastal communities, whereas CRE carriage was low and confined to coastal participants with evidence of clonal transmission. Intestinal carriage of 3GCR-E, including ESBL-producing strains, has been reported to be high in Ghana, with community-based studies showing carriage rates ranging between 30% and 60%, depending on the population and setting (Obeng-Nkrumah et al., 2023; Obeng-Nkrumah et al., 2024). Similar findings of high levels of 3GCR-E have also been documented among clinically relevant isolates with negative impact on patient outcomes (Bediako-Bowan et al., 2020; Labi et al., 2021; Otieku et al., 2023). Environmental studies have likewise reported high levels of antimicrobial-resistant bacteria, particularly ESBL-producing Enterobacterales, in the Korle Lagoon and its tributaries (Banu et al., 2021; Tettey et al., 2024). These observations, together with findings from the present study, confirm the widespread distribution of 3GCR-E in Ghana and its growing threat to the effectiveness of β-lactam antibiotics in treating Gram-negative infections. It also points to the fact that there is likely to be an increase in community acquired infections caused by 3GCR-E in the population.

The main carbapenem resistant genes identified in fecal isolates were *blaOXA-181* and *blaOXA-244*. The *blaOXA*-*181* is an *OXA*-48 like carbapenemase previously reported in Ghana in *E. coli* isolates from the environment and has also been found in clinical *K. pneumoniae* isolates and appears to be a major gene mediating carbapenem resistance among Enterobacterales in Ghana (Labi et al., 2020; Prah et al., 2021; Eger et al., 2024). To the best of our knowledge this is the first time *bla-OXA*-*244* has been reported in Ghana. The *blaOXA-244* has been shown to be prevalent in hospitals in Europe responsible for numerous hospital outbreaks (ASSESSMENT RR, 2021; Grevskott et al., 2024). The presence of OXA-48 like carbapenemases in fecal samples suggests that increasingly these pathogens may be seen in clinical infections such as urinary tract infections. This will pose additional challenges to the healthcare system due to difficulties with laboratory identification (Hoyos-Mallecot et al., 2017; Emeraud et al., 2020) and the limited availability of useful agents for management.

The widespread occurrence of *blaOXA-181* across human and water sources perhaps reflects its integration within highly plastic genetic environments characterized by high replicon diversity and enriched in mobile genetic elements that facilitate horizontal spread. Notably, the ColKP3-type coligenic plasmid was conserved in all *blaOXA-181*–positive isolates, highlighting it’s central role as a vector of carbapenem resistance. ColKP3 plasmids have been widely documented for their strong association with *blaOXA-48–like* carbapenemases and for mediating resistance dissemination across Enterobacterales in both clinical and environmental context (ASSESSMENT RR, 2021; Carfora et al., 2022; Yu et al., 2022; Grevskott et al., 2024; Prah et al., 2025). In our present study, ColKP3 co-occurred with a heterogeneous mix of plasmids whose dominant replicons — IncX3, IncFIB(AP001918), and IncFIA — varied depending on the source. This plasmid repertoire likely enhances compatibility, stability, and persistence within bacterial hosts while enabling synergistic mobilization and horizontal transfer of carbapenem-resistance genes (Yu et al., 2022; Zhang et al., 2023; Prah et al., 2025). Such replicon diversity also provides a versatile genetic platform for recombination and gene exchange, promoting long-term survival of resistance determinants, especially in aquatic environments. The persistence of ColKP3-dominant lineages in aquatic isolates highlights potential of the lagoon environment as a stable, long-term reservoir and dissemination hotspot capable of driving broad interspecies and inter-environmental spread of *blaOXA-181*. Our data raises the concern that the lagoon and its adjoining marine ecosystem could, over time, serve as a reservoir and amplification point for the emergence of CRE endemicity, potentially originating from coastal communities and spreading to the wider human population through interconnected environmental and anthropogenic pathways.

Phylogenetically, *blaOXA-181*–carrying isolates formed mixed clades comprising both human and water strains. This genetic clustering agrees with a shared ancestry and active bidirectional gene flow between environmental and human reservoirs, making it challenging to pinpoint a definitive ultimate source. In contrast, the dissemination pattern of *blaOXA-244* is distinctly different. It was detected predominantly among human isolates and only once in water, with no close genetic relatedness between the two sources. All *blaOXA-244*–positive isolates carried IncY plasmids but lacked the conserved coligenic elements associated with *blaOXA-181*, implying that its spread is mainly clonal within the human population. In this model, *blaOXA-244* water isolates may represent secondary environmental contamination from a human source, although intermittent spillover or reintroduction into the human population cannot be excluded.

Although the cross-sectional design of this study limits the definitive inference of transmission direction, the combined epidemiological evidence provides important insight into the likely dominant flow of CRE between environmental and human reservoirs. The human *blaOXA-181*–positive isolates were genetically more heterogenous (five sequence types, one clade, four singletons) than water isolates, which comprised three sequence types forming distinct ST410, ST940, and ST10 clades. The CRE were persistently detected in nearly all coastal water samples collected over several months, with water isolates displaying high genetic similarity both among themselves and with human isolates. Conversely, human carriage was infrequent (11 carriers among 800 participants), consistent with sporadic a non-endemic acquisition rather than sustained circulation in the community. Antibiotic use among participants from the coastal AMR hotspot community was generally low. Among those with prior antibiotic exposure, carbapenems were unlikely to have been used, as these agents are costly, require parenteral administration, and are not covered by the national health insurance scheme. None of the CRE carriers reported recent hospitalization or travel outside their local communities. Accordingly, CRE prevalence is unlikely to reflect recent antibiotic pressure or external importation but rather local acquisition within the community, an interpretation that aligns well with the multivariable analysis. This suggestion is further buttressed in the multivariable analysis where it is shown that not swimming had a protective effect on the carriage of 3GR-E and ESBL-producers.

Taken together, these findings support the view that the aquatic environment may harbor CRE lineages that acts as a stable resistome reservoir capable of seeding CRE into the human population through exposure pathways such as bathing or fishing. While human carriers inevitably contribute to community spread and reverse contamination of aquatic waters due to poor sanitation practices. The high genomic overlap — evident in the intermixed human and environmental isolates within tight clades (iC1, iC2) — supports a shared, bidirectional resistance gene pool; however, the sustained aquatic presence of CRE, coupled with low human carriage rates, suggests that environment-to-human gene flow may represent the dominant transmission pathway in this setting.

There are some potential limitations to this study. Water sampling did not cover a full annual cycle, and seasonal fluctuations in the lagoon’s water volume may therefore have influenced bacterial concentrations. Furthermore, only 11 water isolates were randomly sequenced from the pool of isolates cultured during surveillance, which may limit the representativeness of the genomic data and the ability to capture the full diversity of environmental strains. The Ashongman inland community, while broadly comparable to the coastal site in socioeconomic and infrastructural characteristics, may nonetheless differ in subtle contextual factors, such as population density, occupational patterns, and other unmeasured factors that could have influenced observed colonization dynamics between the two communities. Despite the study shortcomings, our results provide evidence of human–environmental overlap of resistant *Enterobacterales* in a One Health context and offer genomic data linking coastal waters with community carriage of carbapenemase-producing *E. coli*. Interventions should therefore focus on improving the quality of lagoon water and sanitation practices, including treatment of effluents discharged from multiple sources into the lagoon. Future longitudinal and metagenomic studies are needed to confirm these transmission dynamics.

## Data Availability

The datasets presented in this study can be found in online repositories. The names of the repository/repositories and accession number(s) can be found below: https://www.ncbi.nlm.nih.gov/genbank/, The assembled genomes of the isolates in this study are available in the NCBI GenBank under bioproject accession number PRJNA1297679.
