# Supplementary material for: Human–environmental overlap of resistant Enterobacterales: genomic evidence linking coastal waters and community carriage of antimicrobial resistance in a low- and middle-income setting
Source: Front Antibiot. 2025 Dec 18;4:1715797. doi: 10.3389/frabi.2025.1715797 (PMC12756717; doi:10.3389/frabi.2025.1715797)
Supplement: Supplementary file 1 [file Table1.docx]

| Supplementary Data 1: Distribution of demographic, lifestyle, and environmental variables among coastal and inland cohorts, with prevalence and crude odds ratios (95% CI) for faecal ESBL carriage | | | | | | | | | | | |
| --- | --- | --- | --- | --- | --- | --- | --- | --- | --- | --- | --- |
|  | Coastal | | | | |  | Inland | | | | |
| Variable | Total  (400) | Yes  (188) | No  (212) | Crude OR  (95% CI) | *P* |  | Total  (400) | Yes  (161) | No  (239) | Crude OR  (95% CI) | *P* |
| Gender |  |  |  |  |  |  |  |  |  |  |  |
| Female | 185 | 103 | 82 | 1.92 (1.29–2.86) | 0.0018 |  | 214 | 87 | 127 | 1.04 (0.69–1.55) | 0.92 |
| Male | 215 | 85 | 130 | 0.52 (0.35–0.78) |  |  | 186 | 74 | 112 | 0.96 (0.65–1.44) |  |
| Marital Status |  |  |  |  |  |  |  |  |  |  |  |
| Single | 232 | 111 | 121 | 1.08 (0.73–1.61) | 0.77 |  | 209 | 84 | 125 | 0.99 (0.67–1.48) | 1.00 |
| Married | 146 | 65 | 81 | 0.85 (0.57–1.29) | 0.52 |  | 167 | 66 | 101 | 0.95 (0.63–1.42) | 0.88 |
| Divorced | 9 | 5 | 4 | 1.42 (0.38–5.37) | 0.86 |  | 6 | 2 | 4 | 0.74 (0.13–4.08) | 1.00 |
| Separated | 13 | 7 | 6 | 1.33 (0.44–4.02) | 0.83 |  | 18 | 9 | 9 | 1.51 (0.59–3.90) | 0.54 |
| Alcohol Intake in Past 1 Year | | | |  |  |  |  |  |  |  |  |
| No | 222 | 103 | 119 | 0.95 (0.64–1.41) | 0.87 |  | 281 | 109 | 172 | 0.82 (0.53–1.26) | 0.42 |
| Yes | 178 | 85 | 93 | 1.06 (0.71–1.57) |  |  | 119 | 52 | 67 | 1.22 (0.79–1.89) |  |
| Smoking in Past 3 months | | | |  |  |  |  |  |  |  |  |
| No | 282 | 138 | 144 | 1.11 (0.71–1.74) | 0.64 |  | 390 | 156 | 234 | 1.00 (0.38–2.60) | 1.00 |
| Yes | 118 | 50 | 68 | 0.77 (0.49–1.22) |  |  | 10 | 5 | 5 | 1.50 (0.38–5.91) |  |
| Swimming in Lagoon and Shoreline Waters in Past 1 Year | | | | | | | | |  |  |  |
| No | 40 | 11 | 29 | 0.38 (0.18–0.78) | 0.009 |  | 360 | 136 | 224 | 0.36 (0.19–0.72) | 0.0035 |
| Yes | 360 | 177 | 183 | 2.64 (1.28–5.48) |  |  | 40 | 25 | 15 | 2.75 (1.40–5.39) |  |
| Previous Non-swimming Interactions with Lagoon and Shoreline Waters in Past 1 Year | | | | | | | | | |  |  |
| No | 0 | 0 | 0 | - | - |  | 400 | 161 | 239 | - | - |
| Yes | 400 | 188 | 212 |  |  |  | 0 | 0 | 0 |  |  |
| Handling Raw Meat in Past 1 Year | | | |  |  |  |  |  |  |  |  |
| No | 245 | 103 | 142 | 0.65 (0.43–0.97) | 0.03 |  | 195 | 84 | 111 | 1.26 (0.84–1.90) | 0.26 |
| Yes | 155 | 85 | 70 | 1.67 (1.12–2.51) |  |  | 205 | 77 | 128 | 0.79 (0.53–1.19) |  |
| Handling Raw Fish in Past 1 Year | | | |  |  |  |  |  |  |  |  |
| No | 85 | 37 | 48 | 0.96 (0.56–1.66) | 0.90 |  | 181 | 74 | 107 | 1.05 (0.71–1.56) | 0.81 |
| Yes | 315 | 151 | 164 | 1.19 (0.73–1.93) |  |  | 219 | 87 | 132 | 0.95 (0.64–1.42) |  |
| Source of Drinking Water | | | |  |  |  |  |  |  |  |  |
| Bottled | 5 | 2 | 3 | 1.18 (0.18–7.82) | 0.87 |  | 115 | 42 | 73 | 1.01 (0.62–1.65) | 0.96 |
| Sachet | 345 | 169 | 176 | 1.44 (0.24–8.73) | 0.87 |  | 268 | 103 | 165 | 0.95 (0.63–1.42) | 0.82 |
| Tap | 50 | 25 | 25 | 2.20 (1.05–4.60) | 0.035 |  | 17 | 16 | 1 | 1.05 (0.55–2.02) | 0.88 |
| Mode of Storage of Water (Bathing) | | | |  |  |  |  |  |  |  |  |
| Barrel | 207 | 91 | 116 | 0.84 (0.54–1.29) | 0.42 |  | 107 | 44 | 63 | 1.11 (0.69–1.78) | 0.65 |
| Bucket | 62 | 36 | 26 | 1.77 (0.99–3.13) | 0.05 |  | 25 | 10 | 15 | 0.95 (0.39–2.32) | 0.92 |
| Polytank | 131 | 61 | 70 | 1.11 (0.72–1.72) | 0.64 |  | 268 | 107 | 161 | 1.05 (0.72–1.52) | 0.79 |
| Availability of Toilet Facility | | | |  |  |  |  |  |  |  |  |
| No | 286 | 133 | 153 | 0.93 (0.63–1.38) | 0.75 |  | 21 | 7 | 14 | 0.61 (0.20–1.85) | 0.38 |
| Yes | 114 | 55 | 59 | 1.07 (0.69–1.66) |  |  | 379 | 154 | 225 | 1.64 (0.54–3.47) |  |
| Mode of Waste Disposal | | | |  |  |  |  |  |  |  |  |
| Burning | 23 | 13 | 10 | 0.49 (0.24–0.99) | 0.043 |  | 150 | 60 | 90 | 1.00 (0.65–1.54) | 0.98 |
| Dustbin | 91 | 57 | 34 | 1.83 (0.73–4.60) | 0.20 |  | 230 | 92 | 138 | 0.95 (0.63–1.43) | 0.80 |
| Lagoon/River | 116 | 82 | 34 | 2.63 (1.06–6.54) | 0.04 |  | 4 | 2 | 2 | 1.10 (0.20–5.82) | 0.91 |
| Dumped in pit and burnt | 170 | 36 | 134 | 0.31 (0.13–0.77) | 0.01 |  | 16 | 7 | 9 | 0.88 (0.34–2.28) | 0.79 |
| Hospital Admission | | | |  |  |  |  |  |  |  |  |
| No | 370 | 168 | 202 | 0.42 (0.19–0.91) | 0.03 |  | 382 | 143 | 239 | 0.08 (0.01–0.56) | 0.01 |
| Yes | 30 | 20 | 10 | 2.40 (1.10–5.28) | 0.03 |  | 18 | 18 | 0 | 1.00 (—) | — |
| Antibiotic Use in Past 1 Year | | |  |  |  |  |  |  |  |  |  |
| No | 292 | 137 | 155 | 1.01 (0.65–1.57) | 0.96 |  | 199 | 79 | 120 | 1.12 (0.75–1.65) | 0.62 |
| Yes | 108 | 51 | 57 | 1.01 (0.65–1.57) |  |  | 221 | 82 | 139 | 0.90 (0.60–1.33) |  |
| Travel Outside Locality | | |  |  |  |  |  |  |  |  |  |
| No | 329 | 147 | 182 | 0.59 (0.35–0.99) | 0.05 |  | 325 | 135 | 190 | 1.33 (0.79–2.23) | 0.28 |
| Yes | 71 | 41 | 30 | 1.69 (1.01–2.84) |  |  | 75 | 26 | 49 | 0.75 (0.44–1.26) |  |
| Travel Outside Country | | | |  |  |  |  |  |  |  |  |
| No | 389 | 184 | 205 | 1.21 (0.36–4.08) | 0.77 |  | 376 | 152 | 224 | 0.91 (0.37–2.26) | 0.84 |
| Yes | 11 | 4 | 7 | 0.63 (0.18–2.20) |  |  | 24 | 9 | 15 | 1.10 (0.44–2.76) |  |
| ESBL = extended-spectrum β-lactamase. OR = crude odds ratio. CI = confidence interval. Crude ORs were calculated by comparing each category against all other categories combined within the same cohort (coastal or inland). P-values were derived using Fisher’s exact test. | | | | | | | | | | | |
