# Supplementary material for: Human–environmental overlap of resistant Enterobacterales: genomic evidence linking coastal waters and community carriage of antimicrobial resistance in a low- and middle-income setting
Source: Front Antibiot. 2025 Dec 18;4:1715797. doi: 10.3389/frabi.2025.1715797 (PMC12756717; doi:10.3389/frabi.2025.1715797)
Supplement: Supplementary file 2 [file Table2.docx]

| Supplementary Data 2: Distribution of demographic, lifestyle, and environmental variables among coastal cohorts, with prevalence and crude odds ratios (95% CI) for faecal CRE carriage | | | | | |
| --- | --- | --- | --- | --- | --- |
|  | Coastal | | | | |
| Variable | Total  (400) | Positive CRE carriage (12) | Negative CRE carriage (388) | Crude OR  (95% CI) | *P* |
| Gender |  |  |  |  |  |
| Female | 185 | 9 | 176 | 3.61 (0.96–13.55) | 0.074 |
| Male | 215 | 3 | 212 | 0.28 (0.07–1.04) | - |
| Marital Status |  |  |  |  |  |
| Single | 232 | 7 | 225 | 1.01 (0.32–3.25) | 1.00 |
| Married | 146 | 5 | 141 | 1.25 (0.39–4.02) | 0.765 |
| Divorced | 9 | 0 | 9 | - | 1.00 |
| Separated | 13 | 0 | 13 | - | 1.00 |
| Alcohol Intake in Past 1 Year | | | |  |  |
| No | 222 | 4 | 218 | 0.39 (0.12–1.32 | 0.145 |
| Yes | 178 | 8 | 170 | 2.56 (0.76–8.66) | - |
| Smoking in Past 1 Year | | | |  |  |
| No | 282 | 10 | 272 | 2.13 (0.46–9.88) | 0.522 |
| Yes | 118 | 2 | 116 | 0.47 (0.10–2.17) | - |
| Swimming in Lagoon and Shoreline Waters in Past 1 Year | | | |  |  |
| No | 40 | 0 | 40 | 0.00 (N/A) | 0.619 |
| Yes | 360 | 12 | 348 | *Inf* |  |
| Previous Non-swimming Interactions with Lagoon and Shoreline Waters in Past 1 Year | | | | |  |
| No | 0 | 0 | 0 | N/A | N/A |
| Yes | 400 | 12 | 388 | N/A | N/A |
| Handling Raw Meat in Past 1 Year | | | |  |  |
| No | 245 | 8 | 238 | 1.27 (0.38–4.31) | 0.773 |
| Yes | 155 | 4 | 151 | 0.78 (0.23–2.65) | - |
| Handling Raw Fish in Past 1 Year | | | |  |  |
| No | 85 | 3 | 82 | 1.22 (0.31–4.86) | 1.00 |
| Yes | 315 | 9 | 306 | 0.82 (0.21–3.24) |  |
| Source of Drinking Water | | | |  |  |
| Bottled | 5 | 0 | 5 | 0.00 (N/A) | 0.460 |
| Sachet | 345 | 9 | 336 | 0.73 (0.17–3.19) | 0.665 |
| Tap | 50 | 3 | 47 | 1.88 (0.41–8.68) | 0.409 |
| Mode of Storage of Water (Bathing) | | | |  |  |
| Barrel | 207 | 5 | 202 | 0.65 (0.19–2.24) | 0.773 |
| Bucket | 62 | 3 | 59 | 1.86 (0.49–7.07) | 0.409 |
| Polytank | 131 | 4 | 127 | 1.03 (0.30–3.48) | 1.00 |
| Availability of Toilet Facility at Home | | | |  |  |
| No | 286 | 10 | 276 | 2.03 (0.44–9.41) | 0.522 |
| Yes | 114 | 2 | 112 | 0.49 (0.11–2.29) | - |
| Mode of Waste Disposal | | | |  |  |
| Burning | 23 | 0 | 23 | 0.00 (N/A) | 1.00 |
| Dustbin | 91 | 4 | 87 | 1.73 (0.51–5.88) | 0.481 |
| Lagoon/River | 116 | 5 | 111 | 1.78 (0.55–5.73) | 0.341 |
| Dumped in pit and burnt | 170 | 3 | 167 | 0.44 (0.12–1.65) | 0.250 |
| Hospital Admission | | | |  |  |
| No | 370 | 12 | 358 | *Inf* | 0.612 |
| Yes | 30 | 0 | 30 | 0.00 (N/A) |  |
| Antibiotic Use in Past 1 Year | | |  |  |  |
| No | 292 | 8 | 284 | 0.73 (0.22–2.48) | 0.741 |
| Yes | 108 | 4 | 104 | 1.37 (0.40–4.63) | - |
| Travel Outside Locality | | |  |  |  |
| No | 329 | 12 | 317 | *Inf* | 0.136 |
| Yes | 71 | 0 | 71 | 0.00 (N/A) | - |
| Travel Outside Country | | | |  |  |
| No | 389 | 12 | 377 | *Inf* | 1.00 |
| Yes | 11 | 0 | 11 | 0.00 (N/A) |  |
| CRE = Carbapenemase-producing *E. coli.* OR = crude odds ratio. CI = confidence interval. Infinity (*Inf*) and Not Applicable (N/A) entries for the OR and 95% CI are a result of complete separation of events (i.e., zero CRE-positive cases were observed in one category). ORs were calculated by comparing each category against all other categories combined within the same cohort (coastal or inland). P-values were derived using Fisher’s exact test. | | | | | |
